# Nasal Swab Sampling for SARS-CoV-2: a Convenient Alternative in Times of Nasopharyngeal Swab Shortage

**DOI:** 10.1128/JCM.00721-20

**Published:** 2020-05-26

**Authors:** Hélène Péré, Isabelle Podglajen, Maxime Wack, Edouard Flamarion, Tristan Mirault, Guillaume Goudot, Caroline Hauw-Berlemont, Laetitia Le, Eric Caudron, Sophie Carrabin, Julien Rodary, Tatiana Ribeyre, Laurent Bélec, David Veyer

**Affiliations:** a Laboratoire de Virologie, Hôpital Européen Georges Pompidou, Assistance Publique-Hôpitaux de Paris, Paris, France; b INSERM U970, PARCC, Hôpital Européen Georges Pompidou, Faculté de Médecine, Université de Paris, Paris, France; c Service de Microbiologie, Hôpital Européen Georges Pompidou, Assistance Publique-Hôpitaux de Paris, Paris, France; d Faculté de Médecine Paris Descartes, Sorbonne Paris Cité, Université de Paris, Paris, France; e Département d’Informatique Médicale, Biostatistiques et Santé Publique, Hôpital Européen Georges Pompidou, Assistance Publique-Hôpitaux de Paris, Paris, France; f Centre de Recherche des Cordeliers, INSERM, UMRS 1138, Université de Paris, Paris, France; g Département de Médecine Interne, Hôpital Européen Georges Pompidou, Assistance Publique-Hôpitaux de Paris, Paris, France; h Départment de Médecine Vasculaire, HYPERVASC, Hôpital Européen Georges Pompidou, Assistance Publique-Hôpitaux de Paris, Paris, France; i Service de Médecine Intensive-Réanimation, Hôpital Européen Georges Pompidou, Assistance Publique-Hôpitaux de Paris, Paris, France; j Laboratoire de Chimie Analytique Pharmaceutique, EA7357, Université Paris Sud-Paris-Saclay, Châtenay-Malabry, France; k Départment de Pharmacie, Hôpital Européen Georges Pompidou, Assistance Publique-Hôpitaux de Paris, Paris, France; l Unité de Génomique Fonctionnelle des Tumeurs Solides, Centre de Recherche des Cordeliers, INSERM, Université de Paris, Paris, France; Boston Children’s Hospital

**Keywords:** COVID-19, nasal swab, nasopharyngeal swab, RT-PCR, SARS-CoV-2, molecular diagnosis

## LETTER

Nasopharyngeal swab is the reference sampling method to detect severe acute respiratory syndrome coronavirus 2 (SARS-CoV-2), as recommended by the World Health Organization (WHO) (1). However, nasal specimens may have a slightly lower sensitivity than nasopharyngeal specimens (2, 3). We herein validated an alternative procedure to collect nasal secretions with a swab routinely used in medical bacteriology for which there is no risk of supply disruption in order to perform the molecular diagnosis of SARS-CoV-2 infection.

Patients who were suspected of having coronavirus disease 2019 (COVID-19) attending the Hôpital Européen Georges Pompidou, Paris, France, were for their own care according to medical decision prospectively included and subjected to SARS-CoV-2 molecular testing using nasopharyngeal swab (Xpert nasopharyngeal sample collection kit; Cepheid, Sunnyvale, CA, USA) and nasal swab (Copan Transystem; Copan, Brescia, Italy).

Nasal and nasopharyngeal swabs were inserted in the nostril until they hit an obstacle (the inferior concha and the back of the nasopharyngeal cavity, respectively), rotated five times, and removed. The test was conducted in only one nostril per patient. After sampling, the nasopharyngeal swab was inserted into a vial containing 3 ml of virus transport medium (Xpert viral transport medium; Cepheid), and the nasal swab was placed in a 15-ml tube containing 3 ml of saline solution (0.9% NaCl). SARS CoV-2 was detected using Allplex 2019-nCoV assay (Seegene, Seoul, Korea).

A total of 44 patients were prospectively included up to the end of March 2020. Their median age was 63.0 years, ranging from 18 to 94 years. There were 23 (52.3%) male and 21 female patients. A total of 37 (84.1%) patients showed laboratory-confirmed SARS-CoV-2 infection using nasopharyngeal swab, with 7 patients giving negative results (15.9%) (Table 1).

**TABLE 1:** Comparison of nasopharyngeal versus nasal sampling for SARS-CoV-2 detection by molecular biology

Nasopharyngeal sample/nasal sample results	No. of samples (%)
Concordant results	
Positive/positive	33 (75.0)
Negative/negative	7 (15.9)

Discordant results	
Positive/negative^*a*^	4 (9.1)

Total	44 (100.0)

a Of the four samples with discordant results, two samples had very low viral loads (*C_T_* of 38 on the *N* gene).

Out of 37 patients that were positive for SARS-CoV-2 by nasopharyngeal swab testing, 33 also tested positive by nasal sampling. All SARS-CoV-2-negative patients with nasopharyngeal swabs (*n* = 7) gave negative test results using nasal swabs (Table 1).

By reference to nasopharyngeal sampling, the detection of SARS-CoV-2 by nasal sampling provided 7 (15.9%) true SARS-CoV-2-negative specimens, 4 (9.1%) false-negative specimens, and 33 (75.0%) true SARS-CoV-2-positive specimens. Thus, the sensitivity of SARS-CoV-2 RNA detection by multiplex real-time PCR from nasal secretions was 89.2% (95% confidence interval [95% CI], 75.3 to 95.7), and its specificity was 100.0% (95% CI, 94.6 to 100.0). The κ index was 0.72, indicating substantial concordance between nasal and nasopharyngeal swabbing to detect SARS-CoV-2 according to Landlis and Koch rank. The Youden J index was calculated at 89.2%, demonstrating good efficiency to detect SARS-CoV-2 RNA.

Threshold cycle (*C_T_*) (mean ± standard deviation [SD]) values for *E* (envelope), *RdRP* (RNA-dependent RNA polymerase) and *N* (nucleocapsid) genes by nasopharyngeal (23.9 ± 4.9 for *E*, 26.3 ± 5.5 for *RdRP*, and 28.9 ± 6.1 for *N*) and nasal (22.3 ± 5.2 for *E*, 24.6 ± 5.9 for *RdRP*, and 27.9 ± 6.1 for *N*) swab testing were similar. Differences in *C_T_* values for the *E*, *RdRP*, and *N* genes were not statistically significant (*P* = 0.56, 0.84, and 0.57, respectively).

We herein report that the molecular detection of SARS-CoV-2 using nasal swab specimens was nearly equivalent to the detection using nasopharyngeal swab considered the gold standard. SARS-CoV-2 detection from nasal samples showed high sensitivity and specificity. Agreement and accuracy of test results using nasal sampling by reference to gold standard nasopharyngeal sampling were estimated as substantial and good, respectively. Taken together, these observations demonstrate that nasal sampling could be used to screen SARS-CoV-2 in times of nasopharyngeal swab shortage.
